# Longitudinal associations between treatment adherence patterns and blood pressure control among rural hypertensive patients in Central China: a real-world retrospective cohort study

**DOI:** 10.3389/fmed.2026.1791265

**Published:** 2026-03-25

**Authors:** Bingxi Liu, Yidan Chen, Rao Fu, Nianfang Luo, Xiaobei Wei, Danqing Han, Xiaoxiang Peng

**Affiliations:** 1 School of Medicine, Jianghan University, Wuhan, China; 2 Department of Neurology, Hubei No. 3 People’s Hospital, Jianghan University, Wuhan, China

**Keywords:** blood pressure control, hypertension, longitudinal study, medication adherence, real-world evidence, rural primary care, time in target range

## Abstract

**Background:**

Hypertension remains a major public health challenge in rural China, where sustained blood pressure (BP) control is often suboptimal. Although antihypertensive medication use is central to hypertension management, real-world evidence integrating treatment strategies, medication adherence, and longitudinal BP control in rural primary care settings remains limited. Time in target range (TTR) has emerged as an indicator of sustained blood pressure (BP) control, particularly relevant in community-based settings with infrequent follow-up, yet its application in routine rural primary care management remains insufficiently characterized.

**Methods:**

This real-world retrospective cohort study included 5,029 adults (≥35 years) with diagnosed hypertension who completed four consecutive quarterly follow-up visits in 2024 under China’s National Basic Public Health Service Program in rural Hubei Province. BP control (<140/90 mmHg) was assessed at each visit, and annual TTR was calculated as the proportion of visits meeting the BP target (satisfactory control: TTR ≥ 75%). Medication use (none, monotherapy, combination therapy) and medication adherence (adherent, partially adherent, non-adherent) were integrated into a composite treatment–adherence exposure. Generalized estimating equation models were used to examine longitudinal associations between treatment–adherence patterns and BP control, adjusting for demographic factors, comorbidities, lifestyle variables, and time effects.

**Results:**

Among all participants, 22.1% were not taking antihypertensive medication, 58.5% were on monotherapy, and 19.4% were on combination therapy. Overall BP control rates increased modestly over four quarterly visits. Compared with no medication use, medication use with good adherence was associated with a higher likelihood of BP control [odds ratio (OR) = 1.547, 95% confidence interval (CI): 1.388–1.725], whereas medication use with poor adherence was associated with a lower likelihood of control (OR = 0.847, 95% CI: 0.735–0.975)—suggesting it may be less effective than no treatment, possibly due to irregular intake or unmeasured confounding factors. Longitudinal trends showed stable improvement in BP control among patients with good adherence, while control declined among those not receiving medication. Sex-specific analyses indicated that poor adherence was more adversely associated with BP control among men than women.

**Conclusion:**

In this rural primary care population, sustained antihypertensive medication adherence was consistently associated with better longitudinal BP control, whereas irregular or absent treatment was linked to unstable or declining control. These findings suggest that expanding medication coverage alone may be insufficient for improving hypertension outcomes in rural settings. Routine adherence assessment and targeted strategies for high-risk subgroups, particularly men and patients with diabetes, may help inform community-based hypertension management programs.

## Introduction

Hypertension is one of the most prevalent chronic diseases worldwide, with a continuously increasing prevalence, and has become a major public health problem threatening population health (1). As the most populous country in the world, China faces a particularly prominent burden of hypertension in rural areas. Multiple studies have shown that the prevalence of hypertension in rural China has been increasing year by year, while blood pressure (BP) control rates remain generally low, indicating a concerning status of hypertension management (2, 3). For example, two cross-sectional surveys conducted in Yunnan Province demonstrated that the BP control rate in rural areas increased from 25.8% in 2009 to 30.6% in 2016; despite this improvement, the control rate remained substantially lower than that observed in coastal urban regions (4, 5). In recent years, with the gradual improvement of rural healthcare service systems and the implementation of hypertension prevention and control programs, overall BP control rates among rural hypertensive patients in China have shown an upward trend (6). Correspondingly, the BP control rate among patients under standardized hypertension management increased from 50.88% in 2009 to 67.72% in 2019, with an average annual growth rate of 3.28% (7).

Against this background, indicators used to evaluate BP management have continued to evolve. In recent years, time in target range (TTR) has emerged as a complementary indicator for evaluating the quality of long-term blood pressure management, reflecting both stability and sustainability of control over time (8). While multiple studies have demonstrated an association between higher TTR and reduced cardiovascular risk, and a TTR ≥ 75% is generally recognized as conferring meaningful clinical benefits to patients, the existing evidence is largely derived from clinical trials or cross-sectional studies. Notably, data from real-world rural primary care settings remain limited (9–11). Moreover, most previous studies have assessed antihypertensive medication use without adequately accounting for adherence heterogeneity, potentially obscuring meaningful differences in real-world treatment behaviors. Longitudinal evidence integrating medication strategies, adherence patterns, and repeated blood pressure measurements in community-managed hypertensive populations remains scarce. Hubei Province, a major populous province in central China, faces substantial challenges in the prevention and control of hypertension in rural areas, and the BP control status of hypertensive patients warrants further investigation. In hypertension management, long-term BP control is of critical importance, with lifestyle interventions and pharmacological therapy representing two key strategies; among these, pharmacological treatment serves as the primary therapeutic approach (12). Therefore, investigating the distribution and patterns of antihypertensive medication use is essential for improving hypertension management. Most existing studies focus on cross-sectional assessments of BP control rates, while few have longitudinally examined TTR levels and quarterly BP control dynamics among community-managed hypertensive populations. Accordingly, this study targeted hypertensive patients enrolled in the National Basic Public Health Service program in Lishan Town, Suizhou City, Hubei Province. By collecting BP data from four consecutive quarterly follow-up visits, we aimed to characterize the BP control status throughout the year, as well as the distribution and patterns of commonly used antihypertensive medications among rural hypertensive patients in this region. Furthermore, generalized estimating equation models were applied to analyze the effects of medication adherence and medication strategies on BP control, with the goal of providing empirical evidence to optimize hypertension prevention and control strategies in rural areas.

## Methods

### Study participants and data source

**Figure 1:**
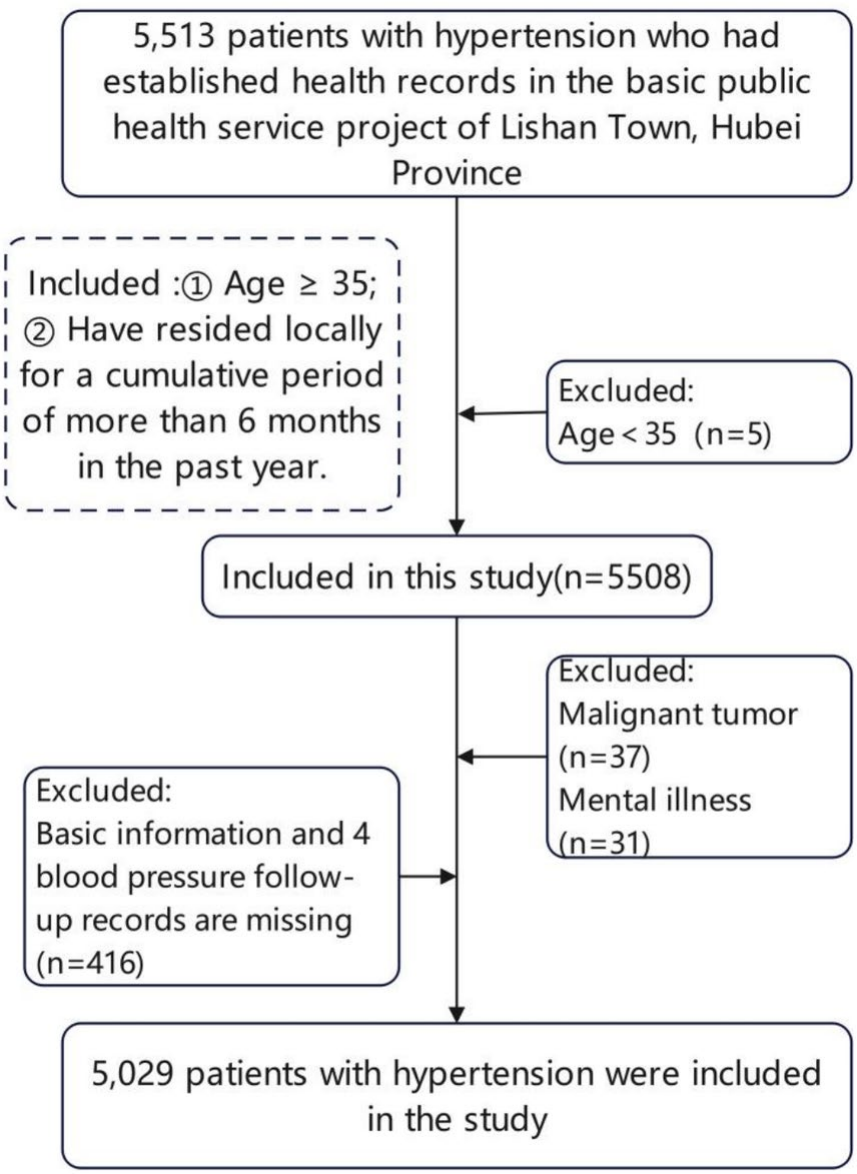
Flow chart of the study population.

From January 2024 to December 2024, we selected 5,513 patients with hypertension who had established health records under the National Basic Public Health Service Program in Lishan Town, Suizhou City, as the study population. Hypertension management in Lishan Town was delivered exclusively through township health centers and village clinics under the National Basic Public Health Service Program. These facilities constitute the primary-level healthcare system in rural China. Township health centers are staffed by licensed physicians and general practitioners and provide outpatient primary care services, whereas village clinics are operated by trained village doctors under the supervision of township health centers. The present study did not include data from secondary or tertiary hospitals. Therefore, the study population reflects community-managed hypertensive patients receiving standardized primary care follow-up rather than hospital-based specialty care. Under the National Basic Public Health Service Program, hypertension management follows nationally standardized protocols, including quarterly follow-up visits, medication assessment, and BP measurement procedures. Although variations in physician experience and local resource availability may exist, the overall management framework and data recording system are uniform across facilities within the township. The inclusion criteria were: (1) age ≥35 years; and (2) residents who had lived cumulatively for more than 6 months in the past year in any of the 26 villages and neighborhood committees of Lishan Town, Suizhou City. The exclusion criteria were: (1) comorbid malignant tumors; (2) comorbid mental disorders; and (3) missing basic information and missing values in the four blood pressure follow-up records. The complete follow-up rate across four quarters was 92.4%, and finally 5,029 patients aged ≥35 years with a prior history of hypertension were included (the flowchart is shown in Figure 1).

### Data collection

The data used in this study were obtained from the information systems of community health service centers in Lishan Town, Suizhou City, specifically from the personal basic information forms of patients with hypertension. The main variables collected included sociodemographic information (sex, age, and whether the individual belonged to the targeted poverty alleviation population), lifestyle factors (smoking, alcohol consumption, and salt intake), educational attainment (university/college, high school, junior high school/primary school, and illiterate or semi-illiterate), history of prior diseases (stroke, coronary heart disease, and diabetes), medication use, medication adherence, and achievement of blood pressure control targets. Information on sociodemographic characteristics, lifestyle, dietary habits, disease history, and medication history was routinely recorded during health examination registration by trained health workers. Baseline characteristics were defined using information collected at the first quarterly follow-up visit during the study period (Q1, 2024), which served as the reference time point for cohort entry.

### Definitions

According to the Chinese Guidelines for the Prevention and Treatment of Hypertension (2024 revised edition), the diagnostic criteria for hypertension were as follows: in the absence of antihypertensive medication use, office blood pressure was measured in a standardized manner on three separate occasions on different days; hypertension was defined as systolic blood pressure (SBP) ≥ 140 mmHg (1 mmHg = 0.133 kPa) and/or diastolic blood pressure (DBP) ≥ 90 mmHg across all three measurements; alternatively, individuals with blood pressure <140/90 mmHg but with a prior history of hypertension were also classified as having hypertension (13, 14). Smoking was defined as smoking ≥1 cigarette/day for ≥1 year, or having quit smoking for <6 months; former smoking was defined as cessation for ≥6 months. Drinking status was categorized into four groups based on alcohol consumption frequency: never (never drinking), occasional (<1 time/week), frequent (≥1 time/week), and daily. Salt intake was classified as mild, moderate, or severe according to perceived saltiness. Diabetes, coronary heart disease, and stroke were defined as current or previous diseases with a clear diagnosis made by a hospital at or above the primary level. Body mass index (BMI) was defined as weight (kg) divided by height (m) squared. Quarterly blood pressure control was defined as both SBP and DBP being <140/90 mmHg among patients with hypertension at the time of the survey. The blood pressure control rate was calculated as the number of participants achieving blood pressure control divided by the total number of participants surveyed × 100%. Satisfactory annual blood pressure control was defined as achieving satisfactory control in ≥3 of the four follow-up visits, i.e., time in target range (TTR) ≥ 75%; TTR (%) = number of follow-up visits meeting the target / total number of follow-up blood pressure measurements × 100% (15). The antihypertensive medications reported by patients were categorized as angiotensin-converting enzyme inhibitors (ACEIs), angiotensin receptor blockers (ARBs), angiotensin receptor–neprilysin inhibitors (ARNIs), β-blockers, calcium channel blockers (CCBs), diuretics, α-blockers, novel single-pill combination formulations, traditional single-pill combination formulations, and traditional Chinese medicine (13, 14). The detailed classification is shown in Supplementary Table S1.

In this study, combination therapy was defined as taking a fixed-dose combination formulation or two or more antihypertensive medications. Medication method was categorized as no antihypertensive medication use, monotherapy, and combination therapy. Medication adherence was assessed using the standardized hypertension follow-up record forms in routine community management. At each quarterly visit, trained health workers documented patients’ current antihypertensive medication-taking behavior as part of the structured follow-up process. Medication adherence was classified as non-adherent, adherent (regular medication use), and partially adherent (intermittent medication use). To address the conceptual inconsistency that adherence cannot be defined among individuals not receiving treatment, medication use and adherence were integrated into a composite treatment–adherence variable. This approach was intended to better reflect real-world treatment behaviors in primary care settings and reduce exposure misclassification. The composite exposure was categorized as (1) no medication use, (2) medication use with poor adherence (non-adherence or intermittent adherence), and (3) medication use with good adherence (regular adherence). The variable coding table is provided in Supplementary Table S2. In routine primary care records, the distinction between non-adherence and intermittent adherence was based on structured follow-up documentation rather than quantitative adherence scales. Given the potential overlap and misclassification between these adjacent categories, and because both patterns reflect insufficient antihypertensive exposure in a quarterly follow-up framework, non-adherent and partially adherent patients were combined into a single “poor adherence” group. This approach aimed to improve exposure stability, enhance model interpretability, and reflect the pragmatic differentiation between sustained versus inconsistent treatment behaviors in real-world community practice.

### Follow-up and blood pressure measurement

According to the National Basic Public Health Service Guidelines (3rd edition), registered hypertensive patients are required to receive standardized follow-up at least once every 3 months. In Lishan Town, follow-up visits were conducted by trained health workers using the structured hypertension follow-up record form under the national public health framework. During each quarterly visit, healthcare providers performed blood pressure measurement, assessed symptoms and comorbid conditions, reviewed medication use and adherence, and provided lifestyle counseling. If blood pressure was not adequately controlled during a visit, treatment adjustment—including dosage modification or addition of antihypertensive agents—could be implemented in accordance with guideline recommendations. Patients with persistently uncontrolled hypertension, significant adverse drug reactions, or clinically complex conditions could be referred to higher-level hospitals for further evaluation and management. Although patients were allowed to seek additional unscheduled outpatient visits when clinically indicated, only the standardized quarterly follow-up records were systematically captured in the public health management database used for this study. The follow-up period was from January 2024 to December 2024. At each quarterly follow-up, trained health workers first documented patients’ antihypertensive medication-taking behavior during the preceding interval, followed by standardized blood pressure measurement at the same visit, ensuring that medication adherence assessment temporally preceded blood pressure evaluation. Blood pressure was measured by qualified physicians in accordance with the guidelines. Participants were first asked to rest for at least 5 min in a quiet room with controllable temperature. Upper-arm blood pressure was then measured using an Omron (HEM-7121) sphygmomanometer in a seated position; three consecutive measurements were taken at 1-min intervals, and the mean of the three readings was recorded as the blood pressure value for that follow-up visit (16).

### Quality control

In this region, multiple approaches were used to ensure data quality for patients with hypertension included in the management program. All follow-up staff were trained professionals who had completed systematic training and passed competency assessments to ensure that data collection was accurate, objective, and standardized. Before data entry, the completeness of follow-up records from primary healthcare institutions was verified, and the logical consistency between blood pressure values and medication records was checked. Data were independently entered by two researchers (Bingxi Liu and Danqing Han), and subsequently reviewed by two other investigators (Xiaobei Wei and Nianfang Luo). Any discrepancies were resolved through discussion or adjudication by a fifth researcher (Xiaoxiang Peng).

### Ethical considerations

This study was approved by the Ethics Committee of Hubei No. 3 People’s Hospital of Jianghan University, the affiliated hospital of Jianghan University (approval No. LW2025017). Informed consent was waived because the analyzed database contained no personally identifiable information. Each participant was assigned a unique code for individual identification. Government-funded health examinations were provided free of charge, and participation was voluntary.

### Statistical analysis

All data were analyzed using R (version 4.4.2). Continuous variables with a normal distribution are presented as mean ± standard deviation (Mean ± SD), and between-group comparisons were performed using the independent-samples *t* test. Continuous variables with a non-normal distribution are presented as median and interquartile range, M (Q_1_, Q_3_), and between-group comparisons were conducted using the Wilcoxon rank-sum test for two independent samples. Categorical variables are presented as *n* (%), and comparisons were performed using the chi-square test or Fisher’s exact test, as appropriate. Generalized estimating equations (GEE) were used to analyze binary outcomes from repeated measurements to account for within-subject correlation across multiple observations. We prioritized model parsimony to maintain statistical stability within the longitudinal GEE framework, particularly given the relatively smaller subgroup sizes when adherence categories were modeled separately. After comparisons using the quasi-likelihood under the independence model criterion (QIC), an exchangeable working correlation structure was selected, as repeated blood pressure measurements within individuals were expected to be correlated and this structure provides robust population-averaged estimates in longitudinal public health studies (Supplementary Table S3). The models adjusted for time effects and all covariates described above, and odds ratios (ORs) with 95% confidence intervals (CIs) were estimated. To examine effect modification by sex, a “treatment–adherence composite × sex” interaction term was first introduced into the main model and evaluated using the Wald test. Subsequently, sex-stratified GEE analyses were conducted to estimate sex-specific effect sizes. The dplyr, geepack, ggplot2, and patchwork packages were used for analyses. As a sensitivity analysis, we modeled satisfactory annual BP control (TTR ≥ 75%) as a person-level binary outcome and fitted multivariable logistic regression models adjusted for age, sex, diabetes, coronary heart disease, stroke, and BMI. A two-sided *p* value <0.05 was considered statistically significant. We acknowledge that as an observational study using routine follow-up data, our GEE models account for within-subject correlation but cannot establish causality. Reverse causality-whereby BP levels at one visit may influence subsequent medication-taking behavior-is a potential limitation inherent to the observational design.

## Results

### General characteristics of the participants

Baseline characteristics of the 5,029 participants stratified by sex are summarized in Table 1. Women (*n* = 2,589) were older than men (*n* = 2,440) and had lower educational attainment. Men reported substantially higher prevalence of smoking and alcohol consumption. Women had a higher prevalence of diabetes, whereas most other clinical characteristics were broadly comparable between sexes. Differences in medication method were observed, with a slightly higher proportion of monotherapy among women. No meaningful sex differences were observed in systolic blood pressure or annual time in target range (TTR) (Table 1).

**Table 1:** Patient demographics and baseline characteristics.

Characteristic	Gender		*p* value
	Female	Male	
	*N* = 2,589	*N* = 2,440	
Age, median (Q1, Q3)	68 (61,75)	66 (59,73)	<0.001
Education, *n* (%)			<0.001
Illiteracy	342 (13.2%)	120 (4.9%)	
Primary school	975 (37.7%)	682 (28.0%)	
Middle school	1,169 (45.2%)	1,431 (58.6%)	
High school	97 (3.7%)	199 (8.2%)	
University or college	6 (0.2%)	8 (0.3%)	
Targeted poverty reduction, *n* (%)			0.097
No	2,447 (94.5%)	2,279 (93.4%)	
Yes	142 (5.5%)	161 (6.6%)	
Diabetes, *n* (%)			<0.001
No	2,074 (80.1%)	2,042 (83.7%)	
Yes	515 (19.9%)	398 (16.3%)	
Coronary heart disease, *n* (%)			0.097
No	2,340 (90.4%)	2,238 (91.7%)	
Yes	249 (9.6%)	202 (8.3%)	
Stroke, *n* (%)			0.946
No	2,505 (96.8%)	2,360 (96.7%)	
Yes	84 (3.2%)	80 (3.3%)	
Smoking, *n* (%)			<0.001
Never	2,560 (98.9%)	1,814 (74.3%)	
Quit smoking	23 (0.9%)	440 (18.0%)	
Smoking	6 (0.2%)	186 (7.6%)	
Drinking, *n* (%)			<0.001
Never	2,345 (90.6%)	1,532 (62.8%)	
Occasional	234 (9.0%)	633 (25.9%)	
Regular	6 (0.2%)	138 (5.7%)	
Everyday	4 (0.2%)	137 (5.6%)	
Salt intake, *n* (%)			0.205
Light	2,104 (81.3%)	1,952 (80.0%)	
Medium	472 (18.2%)	481 (19.7%)	
Heavy	13 (0.5%)	7 (0.3%)	
Drug adherence, *n* (%)			0.079
No	588 (22.7%)	616 (25.2%)	
Partially adherent	311 (12.0%)	301 (12.3%)	
Adherent	1,690 (65.3%)	1,523 (62.4%)	
Medication method, *n* (%)			0.018
Not taking antihypertensive medication	536 (20.7%)	577 (23.6%)	
Monotherapy	1,560 (60.3%)	1,382 (56.6%)	
Combination therapy	493 (19.0%)	481 (19.7%)	
TTR ≥75%, *n* (%)			0.794
No	1,338 (51.7%)	1,252 (51.3%)	
Yes	1,251 (48.3%)	1,188 (48.7%)	
Controlled blood pressure^a^			0.869
No	1,158 (44.7%)	1,097 (45.0%)	
Yes	1,431 (55.3%)	1,343 (55.0%)	
BMI^a^, median (Q1, Q3)	23.81 (22.28, 26.17)	23.94 (22.56, 26.17)	0.015
Heart rate^a^, median (Q1, Q3)	76 (74, 80)	76 (75, 80)	0.010
SBP^a^, median (Q1, Q3)	137 (130, 146)	137 (130, 146)	0.669
DBP^a^, median (Q1, Q3)	82 (77, 87)	82 (78, 87)	0.018

a Baseline blood pressure, BMI, and heart rate; TTR, Time in target range; BMI, Body mass index; DBP, diastolic blood pressure; SBP, systolic blood pressure.

### Blood pressure control across different antihypertensive medication strategies

Among all participants, 1,113 (22.1%) were not taking antihypertensive medication, 2,942 (58.5%) were on monotherapy, and 974 (19.4%) were on combination therapy (Table 2). Blood pressure control rates differed across the four quarterly follow-up visits, with significant between-group differences observed in the second through fourth quarters as well as in annual TTR.

**Table 2:** Comparison of different antihypertensive medication methods on blood pressure control effect.

Characteristic	Medication method			*p* value
	Not taking antihypertensive medication	Monotherapy	Combination therapy	
	*N* = 1,113	*N* = 2,942	*N* = 974	
Controlled blood pressure Q1, *n* (%)				0.608
No	486 (43.7%)	1,335 (45.4%)	434 (44.6%)	
BP control	627 (56.3%)	1,607 (54.6%)	540 (55.4%)	
Controlled blood pressure Q2, *n* (%)				< 0.001
No	646 (58.0%)	1,589 (54.0%)	479 (49.2%)	
BP control	467 (42.0%)	1,353 (46.0%)	495 (50.8%)	
Controlled blood pressure Q3, *n* (%)				<0.001
No	546 (49.1%)	1,171 (39.8%)	348 (35.7%)	
BP control	567 (50.9%)	1,771 (60.2%)	626 (64.3%)	
Controlled blood pressure Q4, *n* (%)				<0.001
No	580 (52.1%)	1,099 (37.4%)	286 (29.4%)	
BP control	533 (47.9%)	1,843 (62.6%)	688 (70.6%)	
TTR≥75%, *n* (%)				<0.001
No	621 (55.8%)	1,506 (51.2%)	463 (47.5%)	
Yes	492 (44.2%)	1,436 (48.8%)	511 (52.5%)	

Q1, Q2, Q3, Q4: represents four quarters. TTR, Time in target range; BP, blood pressure.

In the fourth quarter, blood pressure control was highest in the combination therapy group (70.6%), followed by the monotherapy group (62.6%) and the no-medication group (47.9%) (*p* < 0.001). Annual TTR also differed across medication methods, with the highest proportion of participants achieving TTR ≥ 75% observed in the combination therapy group (52.5%), compared with the monotherapy group (48.8%) and the no-medication group (44.2%) (*p* < 0.001). The blood pressure control rates in the fourth quarter were 55.16, 46.03, 58.94, and 60.93%, respectively, (Supplementary Figure S1), showing an overall trend of first decreasing and then rebounding. These between-group differences are descriptive and do not account for heterogeneity in medication adherence.

### Distribution of antihypertensive medication use

Among participants receiving antihypertensive medication (*n* = 3,916), calcium channel blockers (CCBs) were the most commonly prescribed drug class in both women and men, followed by angiotensin receptor blockers (ARBs) and angiotensin-converting enzyme inhibitors (Supplementary Table S6). Overall prescribing patterns were similar between sexes, although small differences were observed for traditional Chinese medicine use and α-receptor blockers.

### Associations between treatment–adherence patterns and blood pressure control

**Figure 2:**
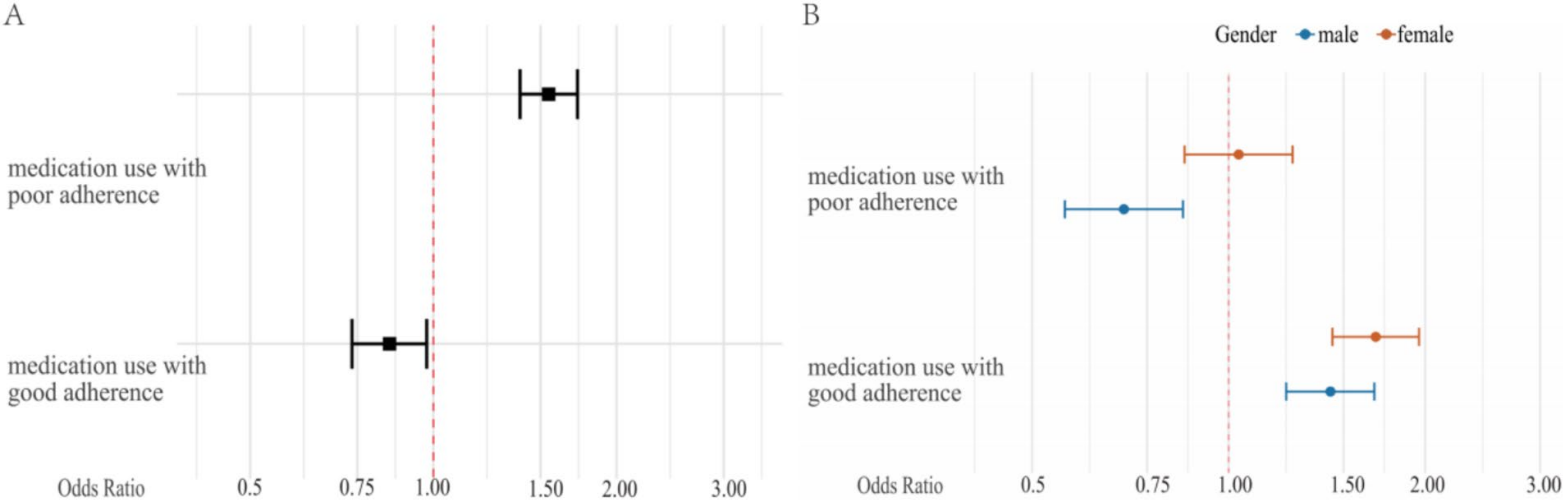
Treatment adherence and blood pressure control: insights and trends. **(A)** The odds ratio for achieving blood pressure control targets associated with the treatment-adherence combination, compared to the no medication use group. **(B)** The effect of the treatment-adherence combination on blood pressure control across gender subgroups: odds ratios and 95% confidence intervals relative to the no medication use group.

Multivariable generalized estimating equation (GEE) analyses accounting for repeated measurements are presented in Table 3. Compared with participants not taking antihypertensive medication, medication use with poor adherence was associated with a lower likelihood of blood pressure control (OR = 0.847, 95% CI: 0.735–0.975; *p* = 0.020), whereas medication use with good adherence was associated with a higher likelihood of control (OR = 1.547, 95% CI: 1.388–1.725; *p* < 0.001) (Figure 2A). Blood pressure control also differed across follow-up visits (OR = 1.138, 95% CI: 1.114–1.162; *p* < 0.001), indicating temporal variation during the study period (Table 3).

**Table 3:** Analysis results of multivariate generalized estimating equations on treatment adherence and blood pressure control in patients with hypertension.

Term	Estimate	Std. err	Wald	OR	95% CI	*p* value
(Intercept)	2.94	0.241	149.24	18.918	11.804–30.32	<0.001
Medication use with poor adherence	−0.167	0.072	5.38	0.847	0.735–0.975	0.020
Medication use with good adherence	0.436	0.055	62.05	1.547	1.388–1.725	<0.001
Time	0.129	0.011	143.38	1.138	1.114–1.162	<0.001
Age	−0.025	0.002	126.76	0.975	0.971–0.979	<0.001
Gender (female vs male)	0.102	0.042	5.92	1.108	1.02–1.203	0.015
Diabetes (yes vs no)	−0.563	0.047	141.48	0.569	0.519–0.625	<0.001
Coronary heart disease (yes vs no)	−0.494	0.074	45.05	0.61	0.528–0.705	<0.001
Stroke (yes vs no)	−0.436	0.120	13.19	0.647	0.511–0.818	<0.001
BMI	−0.061	0.007	78.53	0.941	0.929–0.954	<0.001

Among covariates, comorbid diabetes, older age, higher body mass index, and a history of coronary heart disease or stroke were also associated with lower odds of blood pressure control, whereas female sex showed a modest positive association (Table 3).

### Longitudinal trends in blood pressure control by treatment–adherence pattern

**Figure 3:**
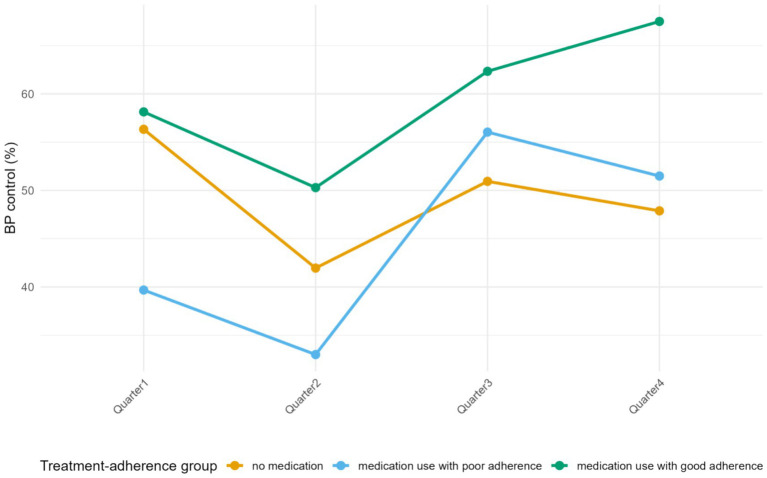
Temporal trends in the rate of blood pressure control.

Distinct trajectories of blood pressure control were observed across treatment–adherence groups over the four quarterly follow-up visits (Figure 3). The medication use with good adherence group showed an increase in control rates from 58.1% in the first quarter to 67.5% in the fourth quarter. In contrast, control rates declined in the no-medication group from 56.3 to 47.9% over the same period. The medication use with poor adherence group (including both non-adherent and intermittently adherent patients) exhibited greater variability in quarterly control rates, including a peak in the third quarter followed by a decline.

### Sex differences in the association between treatment adherence and blood pressure control

As shown in Table 4, a statistically significant interaction between sex and treatment adherence was observed for the comparison of medication use with poor adherence versus no medication (*β* = 0.419, SE = 0.141, Wald = 8.81, *p* = 0.003), indicating that the association between poor adherence and blood pressure control differed by sex. In contrast, the interaction term for medication use with good adherence was not statistically significant (*β* = 0.173, SE = 0.109, Wald = 2.52, *p* = 0.113), suggesting that the beneficial association of good adherence did not significantly differ between men and women.

**Table 4:** Interaction of gender and treatment adherence on blood pressure control.

Characteristic	Estimate	Std. err	Wald	Pr (>|*W*|)
Medication use with poor adherence (female)	0.419	0.141	8.81	0.003
Medication use with good adherence (female)	0.173	0.109	2.52	0.113

Sex-stratified analyses (Supplementary Table S4) showed that medication use with poor adherence was associated with significantly lower odds of BP control in men (OR = 0.69, *p* < 0.001), but not in women (OR = 1.04, *p* = 0.718). A formal test for interaction confirmed that this sex difference was statistically significant (*p* for interaction = 0.003). In contrast, medication use with good adherence was associated with higher odds of control in both men and women, with similar effect sizes and overlapping confidence intervals (Supplementary Table S4; Figure 2B).

### Sensitivity analyses

In sensitivity analyses using satisfactory annual BP control (TTR ≥ 75%) as the person-level outcome, the associations between treatment–adherence patterns and BP control remained consistent with the main GEE results. Compared with no medication use, medication use with poor adherence was associated with significantly lower odds of satisfactory annual control (OR = 0.666, 95% CI 0.543–0.817; *p* = 0.0001), whereas medication use with good adherence was associated with higher odds (OR = 1.472, 95% CI 1.273–1.702; *p* < 0.001) (Supplementary Table S5).

## Discussion

This real-world retrospective cohort study used standardized quarterly follow-up records from China’s National Basic Public Health Service Program to examine longitudinal BP control among 5,029 adults (≥35 years) with hypertension in rural Hubei in 2024. Generalized estimating equations were applied to account for within-individual correlation across repeated BP measurements. To address the conceptual inconsistency that adherence cannot be defined among individuals not receiving antihypertensive treatment, medication strategy and adherence were integrated into a composite treatment–adherence variable, aiming to better reflect real-world treatment behaviors in community primary care settings. Given the observational design, reverse causality represents an important interpretative consideration. Although medication adherence was documented prior to BP measurement at each quarterly visit, our study design does not eliminate the possibility that BP levels observed at one visit may have influenced medication-taking behavior at subsequent visits. For example, patients with persistently uncontrolled BP may become more motivated to adhere to treatment or intensify medication use, whereas others may discontinue therapy due to perceived lack of efficacy, adverse effects, or treatment fatigue. Therefore, while our findings demonstrate a strong longitudinal association between treatment–adherence patterns and subsequent BP control, we cannot rule out reverse causality, whereby BP status may influence future adherence behavior. Consequently, the observed improvement in the “good adherence” group and the decline in the “no medication” group over time may partly reflect dynamic behavioral responses to prior BP levels rather than purely causal effects of adherence itself. Future studies using lagged analytic approaches or prospective intervention designs are warranted to better clarify the temporal directionality of these associations.

The results showed that although the blood pressure control rate at baseline did not differ significantly between the combination therapy group and the monotherapy or not taking antihypertensive medication groups, the combination therapy group exhibited a marked upward trend in blood pressure control over time. Moreover, the proportion of patients achieving blood pressure control within the TTR was highest in the combination therapy group (52.5%), significantly exceeding that observed in the not taking antihypertensive medication group (44.2%) (17). These findings are consistent with current hypertension management guidelines, which emphasize timely treatment intensification and the use of combination therapy strategies for patients with suboptimal blood pressure control (18, 19). Notably, BP control improved modestly over follow-up, and a substantial proportion of participants (22.1%) were not taking antihypertensive medication, highlighting persistent gaps in treatment coverage in rural primary care. Although combination therapy was associated with higher blood pressure control rates at later quarterly visits and with higher annual TTR, these findings should be interpreted with caution, as medication adherence and treatment intensification may differ across treatment groups. The observed treatment gap aligns with findings from other large-scale primary care initiatives, highlighting persistent challenges in the initiation and long-term maintenance of antihypertensive therapy, particularly in resource-limited settings.

Further analyses showed that after multivariable adjustment, treatment–adherence patterns showed clear contrasts: compared with no medication use, poor adherence was associated with a lower likelihood of BP control, whereas good adherence was associated with a higher likelihood of control. Importantly, our findings were robust in sensitivity analyses using satisfactory annual BP control (TTR ≥ 75%) as the outcome, showing consistent associations between treatment–adherence patterns and sustained BP control. These findings suggest that, in routine primary care populations, BP control differs substantially across real-world treatment behaviors, and that medication coverage alone may not adequately capture effective hypertension management. Beyond its use as a study outcome, TTR has important clinical and public health implications. Unlike single-visit BP control rates, which reflect BP status at a specific time point, TTR captures the longitudinal consistency and stability of BP control across follow-up visits. Because cardiovascular risk is more closely associated with cumulative exposure to elevated blood pressure rather than isolated measurements, a higher TTR may better reflect sustained risk reduction in hypertensive populations.

Beyond its use as a study outcome, TTR has important clinical and public health implications. Unlike single-visit BP control rates, which reflect BP status at a specific time point, TTR captures the longitudinal consistency and stability of BP control across follow-up visits. Because cardiovascular risk is more closely associated with cumulative exposure to elevated blood pressure rather than isolated measurements, a higher TTR may better reflect sustained risk reduction in hypertensive populations.

In structured rural primary care settings such as those under the National Basic Public Health Service Program, where ambulatory or home BP monitoring is not routinely available, TTR derived from standardized quarterly visits provides a pragmatic and scalable indicator of ongoing disease control. It may serve as a complementary metric to evaluate the effectiveness of community-based hypertension management programs, reflecting not only pharmacological treatment coverage but also adherence, timely treatment adjustment, and continuity of care. Therefore, the association observed in our study between treatment–adherence patterns and TTR underscores the importance of sustained medication adherence for maintaining longitudinal BP stability. From a public health perspective, strategies that improve adherence and follow-up continuity may contribute not only to improved point-in-time BP control but also to enhanced long-term disease management quality.

Non-adherence is common in hypertension and has been consistently associated with poorer BP control and higher cardiovascular risk in prior studies. In primary care settings with limited consultation time and fewer adherence assessment tools, irregular medication-taking may be insufficiently recognized, potentially complicating treatment decisions and longitudinal BP control (20–23). In primary care settings, where consultation time is limited and adherence assessment tools are often lacking, this problem may be insufficiently recognized, potentially leading to an overestimation of treatment coverage and an underestimation of the true effectiveness of antihypertensive therapy.

The novel composite treatment–adherence variable proposed in this study offers an alternative perspective for understanding how evolving medication-taking patterns influence blood pressure control over time. The longitudinal trend analysis showed that the blood pressure control rate in the medication use with good adherence group increased steadily across follow-up (from 58.1 to 67.5%), whereas the control rate in the no-medication group gradually declined (from 56.3 to 47.9%). In contrast, the medication use with poor adherence group exhibited an unstable, fluctuating pattern without a consistent trend toward improvement. These findings suggest that sustained regular antihypertensive medication use is associated with progressively higher rates of blood pressure control over time (24–27).

Sex-stratified and interaction analyses indicated that the differential association was limited to the medication use with poor adherence category. Specifically, poor adherence was associated with significantly lower odds of BP control in men (OR = 0.69, *p* < 0.001), whereas no statistically significant association was observed in women (OR = 1.04, *p* = 0.718). The interaction term confirmed that this sex difference was statistically significant. In contrast, the beneficial association of good adherence did not significantly differ between men and women, suggesting comparable positive effects across sexes. These findings should be interpreted cautiously. The observed difference reflects a statistically significant interaction for poor adherence rather than uniformly stronger effects in men. The null association among women may relate to differences in baseline characteristics, including higher comorbidity prevalence or variations in treatment patterns, rather than intrinsic biological sex differences. For example, women in this cohort had a slightly higher proportion of monotherapy use than men, which could potentially influence BP control patterns. However, this observation should be considered hypothesis-generating and warrants further investigation in future studies specifically designed to explore sex-based differences in treatment response and adherence dynamics.

Regarding antihypertensive medication selection, CCBs were the most frequently used agents in this population (68.9% in women and 68.4% in men), followed by ARBs (17.7% in women and 19.6% in men). This prescribing profile is consistent with prior real-world prescription studies in Asia and China (19–21), which have consistently reported CCBs and ARBs as among the most commonly used antihypertensive drug classes in Asian populations, with CCBs being particularly prevalent in monotherapy (28, 29). These patterns provide context for rural primary care practice, although our main findings suggest that adherence patterns were more closely aligned with longitudinal BP control than medication strategy alone.

### Strengths and limitations

This study used routine primary care records from a nationally implemented public health framework, with high follow-up completeness and repeated quarterly BP measurements, enabling assessment of within-year BP control dynamics. Second, by defining satisfactory annual control as TTR ≥ 75%, we distinguished short-term attainment from long-term sustained control, thereby providing a more informative indicator of chronic disease management quality. Third, to address the logical inconsistency that adherence cannot be defined for individuals not taking antihypertensive medication, we integrated medication status with adherence to construct a composite treatment–adherence variable, thereby providing an exposure definition that more closely reflects real-world practice and enhances the interpretability of medication-taking behavior patterns. Finally, an exchangeable working correlation structure was selected for the GEE models, consistent with the correlation assumptions inherent to repeated-measures data, and relatively robust effect estimates were obtained after accounting for time effects and multiple covariates.

However, several limitations should be acknowledged. First, as a retrospective study, we could not fully control for all potential confounders, such as behavioral and psychological factors related to medication adherence. Second, the study population was drawn from a single rural region in China, which may limit the generalizability of the findings. Third, Selection bias is also possible because the study required complete quarterly follow-up records; individuals with incomplete follow-up may differ systematically in adherence, access to care, or BP control. Fourth, the study did not include geographic accessibility indicators such as distance to healthcare facilities, transportation availability, or travel time. Although hypertension management was delivered through a geographically organized rural primary care network with assigned village clinics, individual-level access barriers may still influence medication adherence and blood pressure control. The absence of these variables may result in residual confounding. Future studies incorporating geospatial or transportation-related indicators would provide a more comprehensive understanding of access-related disparities in rural hypertension management. Fifth, medication adherence was assessed based on routine follow-up records documented by trained health workers rather than objective measures such as pharmacy refill data or electronic monitoring. Although this approach reflects real-world primary care practice, some degree of misclassification is possible, particularly among patients with intermittent medication use. While TTR ≥ 75% provides a clinically meaningful and pragmatic summary of annual BP control in community-managed populations, our measurement was constrained by the quarterly follow-up schedule. With only four visits per year, TTR can take on only five discrete values (0, 25, 50, 75, 100%), which limits its granularity as an indicator of sustained control. A patient classified as having satisfactory TTR (≥75%) could still have experienced a three-month period of uncontrolled BP between visits. Therefore, TTR in this context should be interpreted as a summary indicator of visit-based control rather than continuous BP stability throughout the year. Future studies incorporating more frequent BP measurements, such as home BP monitoring or digital health technologies, may provide a more refined assessment of sustained BP control and its association with long-term cardiovascular outcomes. Finally, the observation period was limited to one year, precluding evaluation of the long-term sustainability of medication adherence and blood pressure control. Additionally, We recognize that combining non-adherent and partially adherent patients into a single “poor adherence” category may mask potential heterogeneity in blood pressure trajectories within this group. However, given that adherence assessment was based on routine clinical documentation rather than objective or quantitative adherence metrics, separating these categories could introduce instability and overinterpretation of subtle distinctions. Future studies incorporating validated adherence scales or pharmacy refill data should further examine potential dose–response relationships across finer adherence strata. Despite these limitations, the study reflects routine hypertension management under China’s national public health service framework and provides relevant evidence for optimizing population-level strategies in similar rural settings. Future studies could integrate prescription or medication refill data, home blood pressure monitoring or higher-frequency measurements, and longer follow-up to improve the accuracy of adherence and blood pressure control assessment.

These findings highlight that, in rural primary care hypertension management, focusing solely on whether patients use antihypertensive medication may be insufficient; greater emphasis should be placed on long-term adherence and treatment persistence. Targeted, comprehensive management strategies should be explored for high-risk groups. Future research should further evaluate innovative adherence-enhancing interventions, and use longer-term follow-up to elucidate the impact of different treatment–adherence patterns on cardiovascular outcomes.

## Conclusion

Based on longitudinal follow-up data from rural primary care populations in China, this study characterized the dynamic changes in blood pressure control over time among patients with hypertension and highlighted the critical role of treatment adherence. Regular medication use was strongly associated with progressive improvement in blood pressure control, whereas irregular medication use was linked to instability in control. Male sex was associated with less favorable control outcomes under specific treatment–adherence patterns. These findings underscore that improving hypertension outcomes in rural primary care requires more than just expanding medication coverage. Sustained treatment adherence and continuity emerge as critical determinants of long-term blood pressure control. Moreover, integrating routine adherence assessments into community-based follow-up provides real-world evidence to support the implementation of more refined, integrated management strategies for high-risk populations. Prospective studies and intervention trials are warranted to validate these associations and to further optimize hypertension prevention and control strategies in rural settings.

## Data Availability

The raw data supporting the conclusions of this article will be made available by the authors, without undue reservation.
