# Supplementary material for: Longitudinal associations between treatment adherence patterns and blood pressure control among rural hypertensive patients in Central China: a real-world retrospective cohort study
Source: Front Med (Lausanne). 2026 Mar 25;13:1791265. doi: 10.3389/fmed.2026.1791265 (PMC13057556; doi:10.3389/fmed.2026.1791265)

Table S1. Classification of antihypertensive drugs taken by patients.

| Drug Class | | Specific Drugs |
| --- | --- | --- |
| A | ACEI (Angiotensin–Converting Enzyme Inhibitors) | Captopril, Benazepril, Enalapril, Perindopril |
|  | ARB (Angiotensin II Receptor Blockers) | Valsartan, Losartan, Irbesartan, Candesartan, Eprosartan, Telmisartan, Olmesartan |
|  | ARNI(Angiotensin Receptor–Neprilysin Inhibitors) | Sacubitril–Valsartan |
| B | β–Blockers | Metoprolol, Atenolol, Bisoprolol, Labetalol |
| C | CCB (Calcium Channel Blockers) | Nifedipine, Amlodipine, Felodipine, Nimodipine, Lacidipine, Nitrendipine, Isradipine |
| D | Diuretics | Hydrochlorothiazide, Indapamide, Spironolactone, Furosemide |
|  | Single–pill Traditional Combination Formulations | Compound Reserpine, Compound Reserpine–Aminobenzthiazide (Beijing No. 0 Antihypertensive), Compound Robusta, Zhenju Antihypertensive Tablets |
|  | Single–pill New Combination Formulations | Irbesartan–Hydrochlorothiazide, Valsartan–Amlodipine, Amiloride–Combination Tablets, Perindopril–Amlodipine, Olmesartan–Amlodipine |
|  | Traditional Chinese Medicine |  |
|  | α1–Adrenergic Receptor Antagonists | Tamsulosin, Terazosin |

Table S2.Value Assignment Table.

| Variable | Variable Code |
| --- | --- |
| Age | 1: 35–44 2: 45–54 3: 55–64 4: 65–74 5: ≥75 |
| Gender | 0: male 1: female |
| education | 0: illiteracy 1: primary school 2: middle school 3: high school 4: university or college |
| medication method | 0: Not taking antihypertensive medication 1: Monotherapy 2: Combination therapy |
| targeted poverty reduction | 0: no 1: yes |
| Diabetes | 0: no 1: yes |
| Coronary Heart Disease | 0: no 1: yes |
| stroke | 0: no 1: yes |
| smoking | 0: never 1: quit smoking 2: smoking |
| drinking | 0: never 1: Occasional 2: Regular 3: everyday |
| Salt intake | 1: light 2: medium 3: heavy |
| Drug adherence | 0: Non–adherent 1: Intermittent adherent 2: Adherent |
| Treatment–adherence | 0: no medication use 1: medication use with poor adherence 2: medication use with good adherence |

Table S3.The working correlation matrix of the generalized estimation equation.

| structure | QIC | QICu | CIC |
| --- | --- | --- | --- |
| Independence structure | 26478.81 | 26462.74 | 18.04 |
| Exchangeable correlation structure | 26479 | 26463.01 | 18 |
| Autoregressive structure | 26503.86 | 26486.71 | 18.58 |
| Unstructured correlation structure | 26484 | 26468.13 | 17.93 |
| QIC: Quasi–likelihood Information Criterion; QICu: Unscaled QIC; CIC: Conditional Information Criterion. | | | |

Table S4. Effect of treatment adherence on blood pressure control: a gender subgroup analysis.

| Characteristic | female | | male | |
| --- | --- | --- | --- | --- |
| Reference:no medication use | p | OR(95%CI) | p | OR(95%CI） |
| medication use with poor adherence | 0.718 | 1.04 (0.86–1.25) | ＜0.001 | 0.69 (0.56–0.85) |
| medication use with good adherence | ＜0.001 | 1.68 (1.44–1.96) | ＜0.001 | 1.43 (1.23–1.67) |

Table S5. Sensitivity analysis using satisfactory annual BP control (TTR ≥75%) as the outcome.

| **term** | **OR** | **95% CI** | **P value** |
| --- | --- | --- | --- |
| **(Intercept)** | 76.949 | 38.073–155.518 | < 0.001 |
| **Medication use with poor adherence** | 0.666 | 0.543–0.817 | 0.0001 |
| **medication use with good adherence** | 1.472 | 1.273–1.702 | <0.001 |
| **age** | 0.967 | 0.961–0.973 | <0.001 |
| **Gender**  **(Female)** | 1.086 | 0.965–1.221 | 0.171 |
| **Diabetes(yes)** | 0.368 | 0.312–0.433 | <0.001 |
| **Coronary heart disease(yes)** | 0.488 | 0.387–0.617 | <0.001 |
| **Stroke(yes)** | 0.573 | 0.389–0.845 | 0.005 |
| **BMI** | 0.916 | 0.897–0.934 | <0.001 |
| Multivariable logistic regression was fitted with satisfactory annual BP control (TTR ≥75%) as the dependent variable. ORs were adjusted for age, sex, diabetes, coronary heart disease, stroke, and BMI.Reference group: no medication use. | | | |

Table S6. The distribution of medication use among patients with hypertension.

| **Characteristic** | **Taking antihypertensive medication,N=3916** | | **P value** |
| --- | --- | --- | --- |
|  | **Gender(female) N=2,053** | **Gender(male) N=1,863** |  |
| **traditional compound preparations,n(%)** |  |  | 0.756 |
| no | 1,785(86.9%) | 1,626(87.3%) |  |
| yes | 268(13.1%) | 237(12.7%) |  |
| **New compound preparations,n(%)** |  |  | 0.889 |
| no | 2,020(98.4%) | 1,832(98.3%) |  |
| yes | 33(1.6%) | 31(1.7%) |  |
| **Chinese medicine,n(%)** |  |  | 0.032 |
| no | 2,047(99.7%) | 1,863(100.0%) |  |
| yes | 6(0.3%) | 0(0.0%) |  |
| **ARB,n(%)** |  |  | 0.125 |
| no | 1,690(82.3%) | 1,498(80.4%) |  |
| yes | 363(17.7%) | 365(19.6%) |  |
| **ACEI,n(%)** |  |  | 0.207 |
| no | 1,926(93.8%) | 1,729(92.8%) |  |
| yes | 127(6.2%) | 134(7.2%) |  |
| **ARNI,n(%)** |  |  | 0.486 |
| no | 2,046(99.7%) | 1,854(99.5%) |  |
| yes | 7(0.3%) | 9(0.5%) |  |
| **β-receptorblockers,n(%)** |  |  | 0.910 |
| no | 1,970(96.0%) | 1,789(96.0%) |  |
| yes | 83(4.0%) | 74(4.0%) |  |
| **CCB,n(%)** |  |  | 0.744 |
| no | 638(31.1%) | 588(31.6%) |  |
| yes | 1,415(68.9%) | 1,275(68.4%) |  |
| **diuretics,n(%)** |  |  | 0.297 |
| no | 2,007(97.8%) | 1,830(98.2%) |  |
| yes | 46(2.2%) | 33(1.8%) |  |
| **α-receptorblockers,n(%)** |  |  | 0.003 |
| no | 2,053(100.0%) | 1,855(99.6%) |  |
| yes | 0(0.0%) | 8(0.4%) |  |

Figure S1. Overall blood pressure control rate over four quarters.


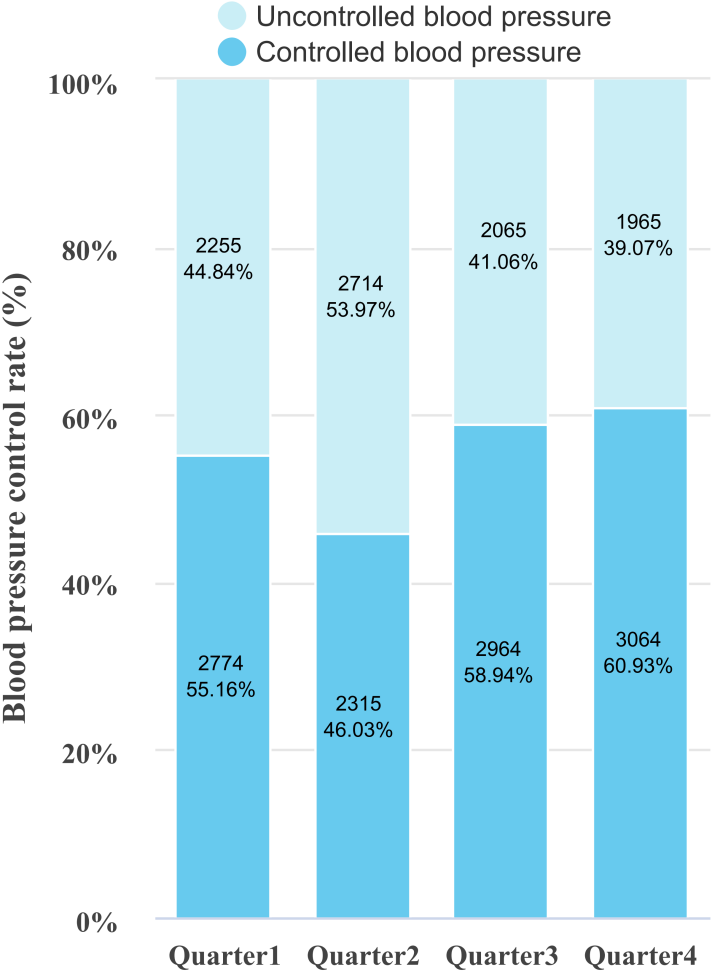

Supplement: Supplementary file 1 [file Table_1.docx]
